# The relationship between social determinants of health and fear of contracting infectious diseases in pregnant women presenting to health centers in Kashan, Iran: a path analysis study

**DOI:** 10.1186/s12888-023-04828-3

**Published:** 2023-05-24

**Authors:** Fatemeh Vakili, Zohreh Mahmoodi, Maliheh Nasiri, Saiedeh Hanieh Alamolhoda

**Affiliations:** 1 Department of Midwifery and Reproductive Health, school of Nursing and Midwifery, Shahid Beheshti University of Medical Sciences, Tehran, Iran; 2 Social Determinants of Health Research Center, Alborz University of Medical Sciences, Karaj, Iran; 3 Department of Basic sciences, Faculty of Nursing and Midwifery, Shahid Beheshti University of Medical Sciences, Tehran, Iran; 4 Department of Midwifery and Reproductive Health, Midwifery and Reproductive Health Research Center, School of Nursing and Midwifery, Shahid Beheshti University of Medical Sciences, Tehran, Iran

**Keywords:** Fear, Infectious disease, Perceived Social Support, Socioeconomic status, Pregnancy-related anxiety

## Abstract

**Introduction:**

Pregnancy is one of the most critical times in a woman’s life that is accompanied by a lot of worry, fear, and stress for the mother, and fear of contracting diseases and losing the children are among the most important of them. The present study investigated the relationship between the social determinants of health and fear of contracting infectious diseases in pregnant women using path analysis.

**Methods:**

This cross-sectional study was conducted on 330 pregnant Iranian women in Kashan from September 21th, 2021, to May 25th, 2022, using a multi-stage method. Data were collected through demographic and obstetric details, fear of COVID-19, perceived social support, socioeconomic status, and pregnancy-related anxiety questionnaires. The collected data were then analyzed using SPSS-21 and Lisrel-8 software.

**Results:**

According to the path analysis results, among the variables that have a causal relationship with fear of contracting infectious diseases through only one path, pregnancy anxiety (B = 0.21) had the highest positive relationship and social support had the highest negative relationship (B=-0.18) in the direct path. Among the variables that have a causal relationship with fear of contracting infectious diseases in both paths, socioeconomic status (B=-0.42) had the highest negative causal relationship with fear of contracting infectious diseases.

**Conclusion:**

According to the path analysis results, the fear of contracting infectious diseases in pregnant women in Kashan is moderate and prevalent, which indicates the necessity of screening pregnant women for such problems during epidemics. Moreover, to prevent this fear and its adverse consequences, the following strategies are recommended: helping promote mothers’ and women’s awareness, offering social support through healthcare providers, and taking measures to mitigate pregnancy-related anxiety in high-risk individuals and groups.

## Introduction

Pregnancy is an important event for women of reproductive age that is associated with significant hormonal, social, psychological, and physiological changes that can make them susceptible to mental illnesses [1–3]. According to reports, mothers have an increased risk of mental-emotional disorders such as depression, anxiety, and disorders related to trauma during this period, especially when the conditions are stressful [4, 5]. During the outbreak of COVID-19, pregnant women have experienced concerns about their own health and that of their unborn babies. As a stressor, this feeling of uncertainty (specificity of an epidemic disease) can increase fear and anxiety in pregnant women [6].

Many studies have discussed the significant psychological impact of COVID-19 on people, which can emerge as fear, panic attacks, and mental health problems [7, 8]. Increased infection-related fear has been reported during many previous epidemics and pandemics and seems to be a common response in all epidemics [9]. In such circumstances, pregnant women are more worried about their older relatives and the health of their children and fetus [10]. They worry about transferring infection to their fetus and the isolation and quarantine of their baby [11].

Disease-related fear and stress lead to symptoms such as changes in sleep and eating patterns, exacerbated psychiatric conditions, and increased substance abuse (e.g., alcohol, tobacco, drugs) [12]. Also, many people reduce or stop their participation in social activities because they fear getting infected [13]. According to reports, fear of infection leads to committing suicide in severe cases [14–16]. More than half of pregnant women experience significant fear and anxiety, and these stressful situations resemble natural disasters and conflicts and can increase the risk of mental health illnesses in the perinatal period [17].

Overall, the cause of this fear is not fully understood; however, a wide range of risk factors have been identified in association with it [18]. This study was conducted to investigate the degree of fear of contracting COVID-19 in pregnant Iranian women and understand the factors related to this problem.

## Materials and methods

### Study type and participants

This cross-sectional study was conducted on 330 pregnant Iranian women visiting comprehensive health centers of Kashan University of Medical Sciences from September 21, 2021, to May 25, 2022.

### Sample size and sampling method

In order for path analysis to achieve an appropriate statistical power, 5–10 samples are required for each parameter [19]. Given that we used path analysis in the current study to investigate the relationship between the variables, the number of questionnaire items on the independent variables (40 parameters) was multiplied by 7. To increase the accuracy by 20% and considering potential sample losses, the sample size was increased to 330.

Pregnant women were selected by multi-stage sampling in such a way that the city of Kashan was divided into five regions (North, South, East, West and Center) and one center was randomly selected from each region. Then, continuous sampling was performed according to the population covered by the selected centers.

### The inclusion criteria were

Married pregnant women aged 18–45 years, reading and writing literacy, no history of mental illness or any known physical illness during pregnancy (according to the self-reports or medical records), no history of COVID-19 during pregnancy, and no history of stressful life events such as death of loved ones during the past six months.

## Data collection

Data were collected using a demographic and obstetric questionnaire, the socioeconomic status questionnaire by Ghodratnama et al., the Pregnancy-related Anxiety Scale (PrAS) by Vandenberg, and the Multidimensional Scale of Perceived Social Support (MSPSS) by Zimet et al. to examine the variables related to the social determinants of health. Since this study was conducted during the COVID-19 pandemic, the seven-item Fear of COVID-19 Scale (FCV-19 S) by Pakpour & Griffiths was used to investigate and measure fear of contracting the disease.

### Demographic and obstetric questionnaire

This questionnaire included items about the mother’s age, husband’s age, ethnicity, gestational age, gravidity, number of children, number of abortions, number of stillbirths, and history of cesarean section.

### Socioeconomic status questionnaire

The socioeconomic status questionnaire consisted of 12 items, five of which were demographic and seven main items inquired about economic class, income, level of education of the individual, level of education of the parents, and housing status. Higher scores denoted higher socioeconomic status. Cronbach’s alpha of this questionnaire was 0.82 according to one study [20].

### Pregnancy-related anxiety scale (PrAS)

PrAS includes 17 items regarding concerns about the health of the fetus, the child’s expenses, the pain of childbirth, etc. The score assigned to each item ranges from 1 to 7. A study by Karamouzian et al. reported the reliability of the questionnaire as Cronbach’s alpha coefficient of 0.78 for the whole scale, and 0.69 to 0.76 for the five factors [21].

### Multidimensional scale of perceived social support (MSPSS)

MSPSS includes 12 items about perceived social support from other people, spouse, friends, and family, and is scored based on a 5-point Likert scale from ‘completely disagree’ to ‘completely agree’. In this scale, ‘completely disagree’ indicates that the person has never received the support discussed in that item, and ‘completely agree’ means that the person has well received the support discussed in that item. Salimi et al. reported Cronbach’s alpha coefficients of 0.89, 0.86 and 0.82 for the three dimensions of social support, i.e., support from the family, friends and important people in life [22].

### Fear of COVID-19 scale (FCV-19 S)

Considering that this study was conducted during the COVID-19 pandemic, we used the seven-item Fear of COVID-19 Scale (FCV-19 S) to examine the concerns and anxieties related to the COVID-19; this scale is scored based on a 5-point Likert scale from ‘completely agree’ to ‘completely disagree’. The lowest and highest scores obtained in this questionnaire are 7 and 35, with higher scores denoting higher fear of contracting COVID-19. The original version had Cronbach’s alpha coefficient of 0.82, retest coefficient of 0.88, and good reliability. Alizadehfard et al. reported Cronbach’s alpha of 0.86 for this scale in Iran [23].

## Ethical considerations

The research started after obtaining permission from the relevant authorities and receiving a code of ethics from the Ethics Committee of Shahid Beheshti University of Medical Sciences (IR.SBMU.PHARMACY.REC.1400.123). First, the researcher visited the selected medical centers and identified eligible pregnant women and then explained the study objectives to them. A written consent form was signed by the women if they were willing to participate in the study. The specified questionnaires were next given to them to complete. A quiet separate space was provided for the mothers to complete the questionnaires in, and if they were not able to complete the questionnaires on the same day, they were asked to complete them within two weeks and to submit them in coordination with the researcher. The researcher’s phone number was given to the mothers to answer any possible questions and doubts. The pregnant women were also assured that their information would remain confidential and there was no compulsion to participate and cooperate in the study and that they would not face any restrictions or problems if they decided not to participate. Continuous sampling was then performed until reaching the required sample size.

### Data analysis

Data were analyzed in SPSS-21 software using descriptive statistics (frequency, mean, standard deviation, and percentage). All variables were quantitatively included in the path analysis; for example, to examine the relationship between education and fear of contracting infectious diseases, the average years of education were included in the path analysis instead of the degree obtained.

**Fig. 1:**
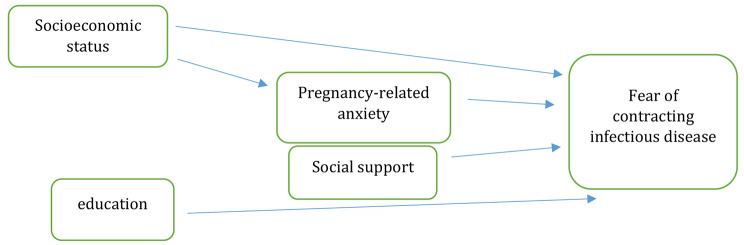
Conceptual model of the relationship between social determinants of health and the fear of contracting an infectious disease in pregnant women

This study assessed the fit of a conceptual model for examining the concurrent effect of social determinants of health with fear of contracting infectious diseases. (Fig. 1). First, the normal distribution of the quantitative variables was assessed using the Kolmogorov-Smirnov test, and then the data were analyzed in SPSS-21, PLS3 [24], and Lisrel-8.8 [25]. In order to test the model, the aforementioned questionnaires were first assessed in the model by PLS. The factor loadings of the items of each questionnaire and the validity and reliability of the tools used in the model were assessed in PLS. According to the results, the factor loadings of all the questionnaires items was higher than 0.4 and all the items were retained after the final testing of the model. To determine the convergent and divergent validity, indices including composite reliability (CR), average variance extracted (AVE), maximum shared variance (MSV), and average shared variance (ASV) were used. The discriminant validity, which means that latent variables that represent different theoretical concepts are statistically different, was assessed by the hetero trait-mono trait ratio of correlations (HTMT); in this measure, values under 0.90 are regarded as having discriminant validity [26].

After assessing the questionnaires in the model we test the model by Path analysis. Path analysis is considered a causal modeling technique; it can be performed with either cross-sectional or longitudinal data and is an extension of the usual regression that shows the direct effects as well as indirect effects and impact of each variable on the dependent variables. All variables in a path model can be described as either endogenous or exogenous. Endogenous variables are diagrammed as being influenced by other variables in the model [27]. The variables diagrammed as independent of any influence are the exogenous variables. Dependent variables are always endogenous, but some independent (or predictor) variables can be endogenous if influenced by other independent variables in the model.

The correlation results were presented as Pearson’s correlation coefficient and the Path analysis results as regression coefficient, Standardized Beta with a significance level of T-value > 1.96.

## Results

### Participants’ characteristics

The data of 330 pregnant women visiting comprehensive health centers of Kashan University of Medical Sciences who remained in the study until the end were analyzed. The mean age of the pregnant women participating in the study and their husband was 30.16 ± 5.46 and 34.65 ± 5.18 years, respectively, and the mean gestational age of the participants was 20 ± 9.33 months. The mean score of socioeconomic status was 13.5 ± 3.72, perceived social support score 22.27 ± 8.79, pregnancy-related anxiety score 56.70 ± 23.66, and fear score was 22.20 ± 7.37 in the participating pregnant women, which indicates high levels of fear in this group (Table 1).

According to the results of Pearson’s correlation test, among the variables that had a significant correlation with fear of contracting infectious diseases in pregnant women, socioeconomic status had the highest negative correlation (r=-0.725, P < 0.001) and pregnancy-related anxiety the highest positive correlation (r = 0.57, P < 0.001). That is, as socioeconomic status improved, fear of contracting infectious diseases decreases, and as pregnancy-related anxiety increases, fear of contracting infectious diseases also increases, and vice versa (Table 2).

**Fig. 2:**
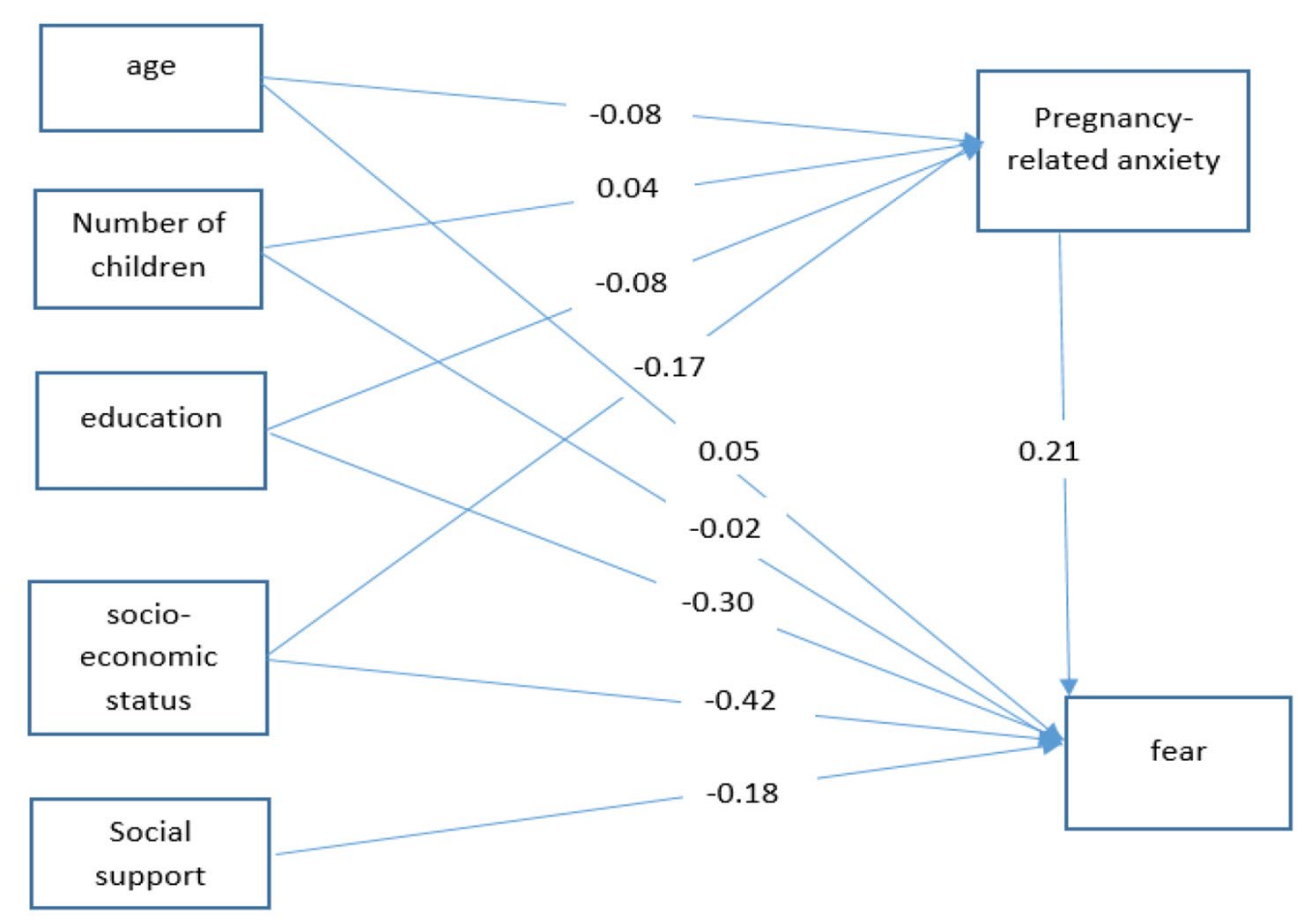
Path analysis test of the relationship between the social determinants of health and fear of contracting infectious diseases in pregnant women

According to the path analysis results, among the variables with a causal relationship with fear of contracting infectious diseases through only one path, pregnancy-related anxiety (B^1^ = 0.21) had the highest positive relationship while education (B=-0.3) had the highest negative relationship in the direct path. Also, perceived social support was negatively related to fear of infectious diseases through the direct path (B=-0.18) (Table 3). In other words, with an increase of one unit in the pregnancy-related anxiety score, fear of contracting infectious diseases increases by 0.21; with an increase of one unit in the education score, the fear score decreases by 0.3; and with an increase of one unit in the social support score, fear of contracting infectious diseases decreases by 0.18. Among the variables with a causal relationship with fear of contracting infectious diseases in both paths, socioeconomic status (B=-0.42) had the highest negative causal relationship with this fear; in other words, socioeconomic status had a negative relationship with pregnancy-related anxiety, and by improving the socioeconomic status, pregnancy-related anxiety decreased and fear of contracting infectious diseases also decreased. Pregnancy-related anxiety thus has a mediating effect on fear of contracting infectious diseases (Figs. 1 and 2).

**Table 1:** Characteristics of pregnant mothers

Quantitative variables	Mean and standard deviation
Mothers’ age	30.16 ± 5.46
Husbands’ age	30.65 ± 5.18
Number of delivery	0.88 ± 0.86
Gestational age	20 ± 9.33
Socioeconomic status	13.5 ± 3.72
Perceived social support	22.72 ± 8.79
Pregnancy-related anxiety	56.70 ± 23.66
Fear of contracting infectious diseases	
Education	
Diploma and lower	136 (%41.2)
Associate Degree	41(%12.4)
Bachelor degree	105(%31.8)
Master and above	48(%14.5)
Job	
Housewife	253(%73.66)
Employed	77(%23.33)
Mother’s age at the birth of the first child	
Younger than 18 years old	13 (%3.96)
18–28 years old	244(%73.93)
29–35 years old	62(%18.87)
Older than 35 years old	11(%3.33)
History of section	
Yes	215 (%65.15)
No	115 (%34.84)

The model fit indices show the desirability and high fit of the model and the reasonableness of the regulated relationships of the variables based on the conceptual model. Accordingly, the fitted model does not have a significant difference with the conceptual model (Table 4).

**Table 2:** The relationship between education, socioeconomic status, pregnancy-related anxiety and perceived social support with fear of contracting infectious disease in pregnant women

Variables	Fear of infectious disease	Education	Socioeconomic status	Pregnancy-related anxiety	Social support	Age	Number of children
Fear of infectious disease	1						
Education	-0.72*	1					
Socioeconomic status	-0.75	0.31*	1				
Pregnancy-related anxiety	0.57*	-0.08	0.01	1			
Social support	-0.35*	-0.07	-0.09	0.01	1		
Age	0.038	0.074	-0.157	-0.055	0.093	1	
Number of children	0.073	-0.221	-0.120	0.080	0.505		1

*: sign of significance

**Table 3:** Path coefficients for variables predicting the fear of contracting infectious disease in pregnant women

Variable	Effect			T-value
	Direct	Indirect	Total	
Socioeconomic status	-0.42*	-0.04*	-0.46*	-3.98
Education	-0.30*	-0.01	-0.31*	-5.27
Pregnancy-related anxiety	0.21*	0	0.21*	7.05
Social support	-0.18*	0	− 0.018*	-4.96
Age	0.05	-0.004	0.046	1.34
Number of children	-0.02	0.008	0.012	0.59

*: sign of significance

**Table 4:** The fit indices of the tested and modified model of social determinants of health and fear of contracting infectious diseases in the studied pregnant women

Indicator	Value	Acceptable value
Chi-Square	0.39	< 3
Degrees of Freedom	1	
Root Mean Square Error of Approximation (RMSEA)	0	< 0.06
Goodness of Fit Index (GFI)	1	> 0.9
Normed Fit Index (NFI)	1	> 0.9
Comparative Fit Index (CFI)	1	> 0.9
Incremental Fit Index (IFI)	1	> 0.9

## Discussion

This study aimed to determine the relationship of fear of contracting infectious diseases with education, socioeconomic status, pregnancy-related anxiety, and perceived social support. The analyses showed that the relationship between fear of contracting infectious diseases and all the above variables is significant, as fear of contracting infectious diseases has a positive relationship with pregnancy-related anxiety and a negative relationship with education, socioeconomic status, and social support.

The mean score of fear of contracting infectious diseases was 22.20 in our study, which is consistent with the research by Naghizadeh et al. in Tabriz, Iran. Their study was conducted to investigate the relationship between fear of COVID-19 and pregnancy-related quality of life during the COVID-19 pandemic [28].

Examining the role of social determinants of health in fear of contracting infectious diseases in pregnant women led us to a significant and negative relationship between education level and fear of contracting infectious diseases. This finding is in line with the study conducted by Doshi et al. in India that aimed to investigate fear of COVID-19 in the Indian population [29].

Based on the findings of the path analysis, pregnancy-related anxiety had a causal and positive relationship with fear of contracting infectious diseases directly and only through one path. During pandemics, including the COVID-19 pandemic, pregnant women feel afraid of contracting an illness themselves or in their family when visiting hospitals, and because of this stress and anxiety, they might want to end their pregnancy early or by cesarean section. During a pandemic, pregnant women might feel fear and anxiety about the health of their fetus and themselves [23]. Pregnant women tend to believe that they are more likely to have a severe episode of infection and may transmit the infection to their unborn child [30]. A study conducted in the United States showed that anxiety and depression were the most frequently-reported mental health issues during the pandemic that affected mainly women, especially pregnant and postpartum women [31]. Lifestyle changes due to COVID-19 infection impose a mental burden on pregnant women, who experience more anxiety and uncertainty than ever during this important time in their life [32], and their anxiety levels can thus rise and more fear of contracting infectious diseases can develop in them.

According to another finding of this study, socioeconomic status is related to fear of infectious diseases in both direct and indirect paths. The present findings are consistent with the global trend on this subject, which has shown that the populations most at risk during pandemics are those with lower incomes and less education compared to those who have better housing conditions and can stay home and continue their work from home [33, 34]. In addition, correlations have been shown in several countries between state-level income inequality and COVID-19 cases and deaths, including in Italy and the US, according to different studies [35, 36]. To improve preventive behaviors in this vulnerable group, interventions should be designed and implemented that target low-income and low-educated people [37].

Social support was another variable that had a direct, negative, causal relationship with fear of infectious diseases, and this finding is in line with the results of other studies suggesting a negative relationship between social support and mental health problems [10, 37, 38]. Also, this finding is in line with the study conducted by Muyor-Rodríguez et al. in Spain with the aim of investigating the factors affecting fear of COVID-19 [39]. A possible explanation for this relationship is that social support causes positive feelings and happiness, helps people cope with stress, provides the needed information and assistance, and creates a sense of worth in people that increases their self-esteem and self-sufficiency [40, 41].

Our study also had limitations, mainly that it was conducted on people living in a single city, i.e., Kashan, who are of Fars ethnicity and could have differences with other ethnicities; therefore, we recommend to conduct further studies in other ethnicities and to compare the results with each other. Another limitation of this study was the use of the Fear of COVID-19 Scale to investigate fear of contracting infectious diseases, which makes it difficult to generalize the results to other infectious diseases; therefore, more studies are recommended to be conducted in the future on other common infectious diseases.

## Conclusion

The results of this study revealed the high prevalence of fear of contracting infectious diseases and its relationship with education, socioeconomic status, pregnancy-related anxiety, and perceived social support in pregnant women referring to comprehensive health centers in Kashan.

Pregnant women visit comprehensive health centers to receive care, and should be effectively screened by healthcare workers for fear of contracting infectious diseases during sensitive times and the necessary measures and interventions should be adopted in these high-risk groups because such fear not only affects the quality of life of the pregnant women, but can also affect the quality of life of the other family members and how the women receive healthcare services.

## Data Availability

The datasets used and/or analyzed during the current study are available from the corresponding author on reasonable request.

## Footnotes

1 Beta coefficient.
